# Editorial: Medical physics preparation and certification

**DOI:** 10.1120/jacmp.v13i4.4009

**Published:** 2012-07-05

September 30 of this year marks the last time that a medical physicist seeking certification by the American Board of Radiology may apply for the examination without having completed an accredited clinical training program. The apparent imbalance between individuals seeking such clinical training and the number of available positions in accredited clinical residency programs has been the subject of significant concern among medical physicists in North America. Recently, George Sherouse, PhD, a practicing medical physicist in Charlotte, NC with over 30 years of experience, posted some of his reflections regarding the upcoming imbalance. I have asked and received Dr Sherouse's permission to share his reflections with the *Journal of Applied Clinical Medical Physics* community, in the following guest editorial. As always, comments are most welcome.

George Starkschall, PhD

Editor‐in‐Chief

July 5, 2012

